# The state of the art in medical therapies for pediatric heart failure

**DOI:** 10.1016/j.jhlto.2025.100292

**Published:** 2025-05-29

**Authors:** Humera Ahmed, Joseph W. Rossano

**Affiliations:** Division of Cardiology at the Children’s Hospital of Philadelphia and the University of Pennsylvania School of Medicine, Philadelphia, Pennsylvania

**Keywords:** pediatric cardiology, heart failure, clinical trials, pharmacotherapy, gene therapy, cardiomyopathy, congenital heart disease

## Abstract

Pediatric heart failure is a rare but serious condition affecting children with congenital heart disease and various forms of cardiomyopathy. The treatment paradigm for pediatric heart failure has historically been shaped by expert consensus guidelines, largely informed by the results of adult heart failure trials. Recently, however, there has been an increased focus on pediatric-specific drug development and clinical trials. Medications such as digoxin, beta-blockers, and renin-angiotensin-aldosterone system inhibitors have been explored in children, but responses can vary based on the underlying heart disease. Newer treatments such as sacubitril-valsartan and sodium-glucose cotransporter 2 inhibitors show promise, but more data are needed to determine their safety and efficacy in young children. This article explores the current state of medical therapy for chronic pediatric heart failure, highlighting the evolution of treatment strategies and the novel therapies under exploration.

## Background

Pediatric heart failure (HF) is a complex clinical and pathophysiological condition affecting children with congenital heart disease (CHD) and cardiomyopathies.^1^ While the incidence is relatively rare, estimated at 0.9 to 7.4 per 100,000, it is associated with significant morbidity and mortality.^2^, ^3^, ^4^, ^5^, ^6^ Although infants comprise approximately 65% of HF hospitalizations among individuals ≤18 years of age, the paradigm for the treatment of pediatric HF has historically relied on the extrapolation of data from trials performed in adults.^2^, ^7^ This is due to the heterogeneity and relative rarity of pediatric HF, which disincentives the conduct of costly trials in this population and hinders the ability to adequately power and recruit for studies. Fortunately, things have begun to shift over the last decade, following the enactment of legislation incentivizing orphan disease research and pediatric-specific labeling at the time that a new product enters the market, and the creation of multicenter quality improvement initiatives.^8^, ^9^ As a result, there has been a growth in novel pediatric drug trials and pediatric labeling of existing HF therapies. This article reflects on the current state of medical therapy for chronic pediatric HF.

### The cornerstones of pediatric HF pharmacotherapy

#### Digoxin

Digoxin played a central role in the treatment of HF for many decades due to its beneficial antiarrhythmic effect, increased myocardial contractility, and lowering of heart rate (HR). Its use decreased following several posthoc analyses of major adult HF trials that revealed an increased frequency of arrhythmias and hypotension, and reduced left ventricular ejection fraction (LVEF), in patients who were on digoxin at the time of enrollment in the study, when compared to those who were not.^10^, ^11^, ^12^ However, these comparisons did not account for a potential difference in the severity of illness between the 2 groups. Because digoxin is commonly used as a second-line agent for HF, those patients on digoxin at enrollment may have been sicker at baseline. Meta-analyses suggest a more favorable effect of the use of digoxin in adult HF, revealing an association between its use and a 14% relative risk reduction in all-cause mortality^13^ and an 8% reduction in all-cause hospital admissions.^14^

Several retrospective pediatric database studies suggest that digoxin use during the interstage period could offer a survival advantage for patients with single ventricle CHD (SVCHD^15^, ^16^, ^17^, ^18^). Some speculate that the benefits may be linked to the inotropic and antiarrhythmic properties of digoxin, while others propose that the survival advantage could result from a reduction in HR, leading to increased diastolic filling time and enhanced coronary perfusion.

It is important to note that digoxin has a narrow therapeutic window and therefore requires close monitoring to avoid toxicity. A level should be drawn 6 to 8 hours after the last dose, targeting a level of 0.5 to 0.9 ng/ml for HF. The highest risk of toxicity occurs at levels >2 ng/ml, but can occur at lower levels in the setting of electrolyte abnormalities such as hypokalemia. Its use should be avoided in patients with abnormal atrioventricular conduction.

#### Beta-blockers

β-Adrenergic receptor blockers are a mainstay of cardiac reverse remodeling in adults with HF and have been demonstrated to improve symptoms and survival.^19^, ^20^ When carvedilol was studied in a multicenter, randomized, double-blind, placebo-controlled trial of 161 children and adolescents with symptomatic systolic HF, however, there was no difference in HF symptoms, mortality, or hospitalizations compared to placebo.^21^ This may be due to underpowering of the study, underdosing, a heterogenous study population, and the inherent differences in the pathophysiology of adult and pediatric HF.^22^, ^23^, ^24^ Molecular studies have revealed that while adults in HF primarily exhibit a downregulation of β2-adrenergic receptors, children seem to downregulate both β1- and β2-adrenergic receptors; the impact of this differential expression of β-receptors on the efficacy of beta-blockers has not been studied.^25^

#### Inhibitors of the renin-angiotensin-aldosterone system

Angiotensin-converting enzyme inhibitors (ACEis) are associated with symptomatic improvement, reduced progression of HF, decreased hospitalization, and improved survival in adults with HF.^26^, ^27^, ^28^, ^29^ Pediatric trials of this class of mediation have been small and seem to indicate a differential benefit based on the underlying etiology of HF. In a retrospective review of 81 children with dilated cardiomyopathy and systolic ventricular dysfunction, those children who received an ACEi had greater survival at 1 year of follow-up, and a trend toward improved survival at 2 years, when compared to patients who only received “conventional” therapy—consisting of digoxin, diuretics, and spironolactone.^30^ The benefits were amplified in a prospective, randomized, double-blind, placebo-controlled trial of perindopril in boys with Duchenne muscular dystrophy (DMD) between the ages of 9.5 and 13 years with preserved ventricular function, which revealed a 27.4% absolute risk reduction in all-cause mortality at 10 years for patients in the perindopril group.^31^

The results have not been as favorable in the SVCHD population. The largest randomized, double-blind, placebo-controlled trial of enalapril was performed in 230 infants (mean age 20 days) with SVCHD.^32^ At 14 months of follow-up, there was no difference between the groups in somatic growth, ventricular function, beta natriuretic peptide, or the incidence of heart transplantation or death. When studied in a double-blind crossover trial of 18 Fontan patients with a mean age of 14.5 ± 6.2 years, there was no improvement in exercise capacity, systemic vascular resistance, resting cardiac index, or diastolic function after 10 weeks of treatment.^33^

The angiotensin receptor blockers (ARBs) are used primarily in children who are not tolerant of ACEis. In a prospective, randomized, placebo-controlled trial of 5,000 adults with HF, valsartan resulted in significant improvements in New York Heart Association (NYHA) class, ejection fraction (EF), signs and symptoms of HF, and quality of life as compared with placebo. When studied in children, there appears to be a differential response to ARBs based on the underlying etiology of HF. In a randomized trial comparing lisinopril (5 mg/day) and losartan (25 mg/day) in 22 boys with DMD, there was a significant improvement in LVEF that was sustained at 1 year, without a difference between the 2 groups.^34^ Conversely, a double-blind, randomized, placebo-controlled pilot study in a group of young adults with a systemic right ventricle showed that there was no difference between placebo and valsartan in right ventricular EF, exercise capacity, or quality of life.^35^

Spironolactone was studied in a prospective, randomized, double-blind, placebo-controlled study of 1,663 adults with severe HF and a LVEF <35%, who were already being treated with an ACEi and loop diuretic ± digoxin.^36^ The trial was terminated early due to a 30% reduction in the risk of death in the spironolactone group, improvement in HF symptoms, and 35% fewer hospitalizations. The incidence of serious hyperkalemia was minimal in both groups. Studies of spironolactone in pediatric HF are limited. Its use was examined in a study of 21 infants <1 year old with HF secondary to CHD who were treated with digoxin and chlorothiazide; half received additional therapy with spironolactone and the other half with potassium.^37^ Infants treated with spironolactone had a significant reduction in objective measures of volume overload and a slight reduction in HF symptoms. A newer, nonsteroidal mineralocorticoid receptor antagonist, Finerenone, demonstrated a significantly lower rate of total worsening HF and death from cardiovascular causes in adults with preserved or mildly reduced EF; no pediatric studies have been completed to date.

### Newer HF therapies

#### Angiotensin receptor/neprilysin inhibitor

In the 2020 revision of the American College of Cardiology and American Heart Association HF guidelines, angiotensin receptor-neprilysin inhibitors replaced ACE inhibitors and ARBs as the recommended first-line treatment for HF with reduced EF.^38^ This recommendation was primarily based on the outcomes of the PARADIGM-HF trial, a randomized, double-blind study involving 8,442 patients with class II to IV HF and a LVEF of ≤40%.^39^ The trial compared sacubitril-valsartan 200 mg twice daily (BID) to enalapril 10 mg BID and was halted early after meeting prespecified criteria for superiority—there was a 16% reduction in all-cause mortality, a 21% reduction in HF hospitalizations, and fewer symptoms and physical limitations in patients treated with sacubitril-valsartan compared to those on enalapril. Although a higher percentage of patients on sacubitril-valsartan experienced hypotension and mild angioedema, fewer patients experienced renal complications, hyperkalemia, or cough. Sacubitril-valsartan was not superior to valsartan alone in adult patients with HF with preserved EF.^40^

PANORAMA-HF is the pediatric correlate to the PARADIGM-HF trial.^41^ The study included children with HF, divided into 3 age groups at randomization (6 to <18 years, 1 to <6 years, and 1 month to <1 year) and classified by functional status (NYHA/Ross class I/II or III/IV). Sacubitril-valsartan was dosed at 2.3 mg/kg BID for infants and 3.1 mg/kg BID for the older children, while enalapril was dosed at 0.15 mg/kg BID for the youngest group and 0.2 mg/kg BID for the others. The study included both patients with cardiomyopathy and CHD, but excluded those with SVCHD, restrictive cardiomyopathy, hypertrophic cardiomyopathy (HCM), or a systemic right ventricle. Due to the challenges of adequately powering the study using traditional HF outcomes such as mortality and hospitalization, the trial incorporated a novel primary end-point based on a global rank score. Food and Drug Administration (FDA) approval was granted after an interim analysis at 12 weeks showed that sacubitril-valsartan led to a 44% reduction in N-terminal probrain natriuretic peptide (NT-proBNP), which was larger than what was observed in the enalapril group (33% reduction) and exceeded the results of the PARADIGM-HF trial. At study completion, sacubitril-valsartan was not superior to enalapril in either the global rank score or its individual components, but both treatment groups experienced significant improvements in HF classification, patient-reported outcomes, and NT-proBNP.

There are limited data on the real-world use of sacubitril-valsartan in pediatric patients. Its adoption has been limited by cost and challenges with insurance approval, the need for a compounding pharmacy, limited pediatric familiarity with the drug, and tolerance—namely hypotension. A retrospective, single-center report of 23 pediatric patients with dilated cardiomyopathy and left ventricular systolic dysfunction who were treated with sacubitril-valsartan revealed a few practical considerations for the use of sacubitril-valsartan in pediatrics.^42^ Patients had been initiated on sacubitril-valsartan if they were >1 month old, had an LVEF <55%, and had failed to improve after 12 months of standard HF therapy. Given reports of medication intolerance in young patients due to hypotension, sacubitril-valsartan was initiated at a dose of 0.2 mg/kg BID and increased by 0.1 mg/kg every 3 days; both of which are much lower than what was used in the PANORAMA-HF study. If the blood pressure remained >70/50 mm Hg, the dose was increased as tolerated to the age-based goal doses established in the PANORAMA-HF study. The mean maintenance dose achieved in the cohort was 1.84 ± 0.82 mg/kg/day. Despite tolerating less than half of the established target dose, most patients improved after 3 months of therapy, and improvement was sustained at 6 months. The mean LVEF increased from 38% to 52%, left ventricular size decreased from 4.6 to 4.5 cm, and log-transformed serum NT-proBNP decreased by 5.38 at 6 months. All patients (*n* = 11) with a baseline LVEF <40%, and 10 of 12 (83%) with LVEF >40%, demonstrated a ≥10% improvement in LVEF at 6 months of follow-up. Notably, there was no mention of adverse events in the group and no report of the impact of sacubitril-valsartan on electrolytes or estimated glomerular filtration rate.

#### Sodium-glucose cotransporter 2 inhibitors

**Figure 1:**
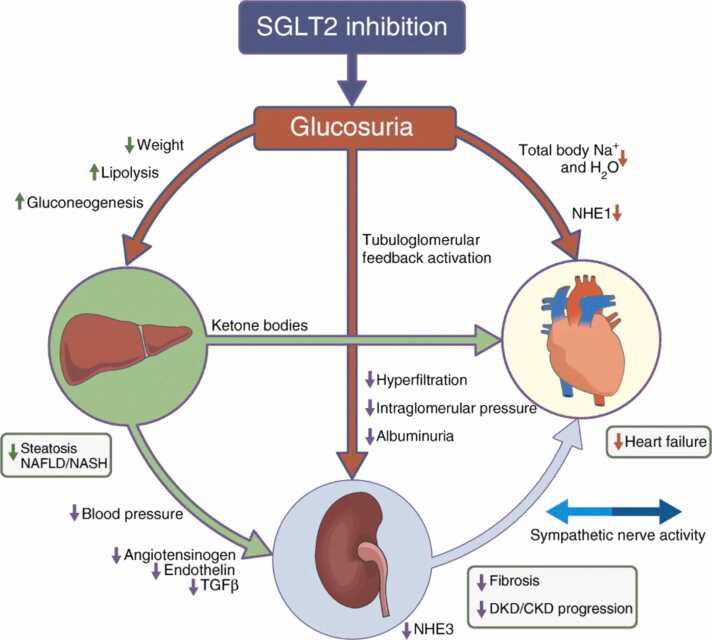
The mechanism of heart failure modulation with the use of SGLT2i. The exact mechanism of heart failure modulation with the use of sodium-glucose cotransporter 2 inhibitors is not well known, but is postulated to be multifactorial.^1^ Glucosuria results in decreased weight, decreased serum glucose with a potential resultant decrease in inflammation, and increased lipolysis, all of which improve cardiometabolic health.^2^ Glucosuria stimulates osmotic diuresis, leading to augmented clearance of intercellular free water.^3^ NHE1 inhibition may reduce intracellular sodium load and restore cardiac mitochondrial function.^4^ Decreased intraglomerular pressure and decreased hyperfiltration may decrease the effects of cardiorenal syndrome. CKD, chronic kidney disease; NHE1, sarcolemmal Na+/H+ exchanger isoform 1; NHE3, tubular Na+/H+ exchanger isoform 3; DKD, diabetic kidney disease.

In the latest revision of the HF guidelines by the American College of Cardiology and the American Heart Association, sodium-glucose cotransporter 2 inhibitors (SGLT2i) were included in guidelines-directed medical therapy for HF with reduced, mildly reduced, and preserved EF.^43^ This inclusion is based on multiple studies that demonstrate decreased risk of HF-related hospitalizations and mortality in adults, in addition to known benefits for type 2 diabetes and chronic kidney disease (Figure 1).^44^, ^45^, ^46^, ^47^

Image permission requested and pending from Springer Nature (Wanner and Marx^48^).

While there have been no prospective trials specifically evaluating SGLT2i use in pediatric HF, empagliflozin and dapagliflozin have both received FDA approval for treating type 2 diabetes in children ≥10 years old.^49^, ^50^ In the largest published retrospective analysis of SGLT2i in pediatric HF, a dose range of 0.1 to 0.2 mg/kg once daily (maximum: 10 mg) was administered to 38 nondiabetic patients with HF.^51^ The median age of the group was 12.2 years (60.5% were 5-17 years and 5.3% were ≤1 year old). Compared to baseline, the EF increased from 32% to 37%, the number of patients with EF >40% increased from 1 to 7 (*p* = 0.04), and B-type natriuretic peptide (BNP) decreased from 222 to 166 pg/ml (*p* = 0.04); notably, these improvements may be partially attributed to medication initiation during a HF exacerbation. There were no significant changes in vital signs, serum electrolytes, estimated glomerular filtration rate, or the use of other concomitant medications. Four patients experienced acute kidney injury and 6 had symptomatic urinary tract infections.

Data on patients with SVCHD are limited. A retrospective case series involving 14 patients treated with an SGLT2i showed an improvement in serum natriuretic peptide levels without incidents of genitourinary infections, hypoglycemia, ketoacidosis, hypotension, or other notable adverse effects.^52^ The ACTION network of pediatric HF centers has compiled data from 19 centers on SGLT2i use, which includes >250 pediatric patients; preliminary analyses suggest medication tolerance without significant adverse effects in short-term follow-up.

Given the insights gained from landmark pediatric HF trials, there is a strong need for a prospective study on the use of SGLT2i in pediatric HF. Such a trial should include pharmacokinetic and pharmacodynamic data for children under 6 years old, as SGLT2i dosing has not yet been established for this age group. In a study of dapagliflozin in children aged 10 to 17 years, the maximum concentration (tmax) occurred around 1.5 hours, and the half-life ranged from 10 to 14 hours.^53^ The area under the curve and urinary glucose excretion were dose-proportional, and all groups showed a reduction in fasting plasma glucose levels. A model developed for pediatric patients with diabetes suggested that the adult dose of 10 mg is suitable for children aged 13 to 18 years, while a 5-mg dose is appropriate for those aged 6 to 12 years.^54^ Common side effects observed in the pediatric population included urinary tract infections, headaches, pharyngitis, vomiting, and hyperglycemia. There is a rare risk of diabetic ketoacidosis, as the body may increase lipolysis during states of fasting or illness.^55^ Importantly, there are no data on how the inhibition of glucose reabsorption affects neurodevelopment in infants and young children.

#### Ivabradine

A randomized, double-blind, placebo-controlled trial assessing ivabradine in adults with severe symptomatic HF (EF <35%) demonstrated a ∼25% relative reduction in the composite outcome of HF-related hospitalizations and mortality in the treatment group.^56^ Additional subgroup analyses suggested that HR reduction could act as a surrogate marker for the primary outcome, with the therapeutic effect of ivabradine primarily stemming from its ability to lower HR.^57^ In the placebo group, patients with the highest baseline HRs (≥87 bpm) experienced a 2.34-fold increase in the risk of the primary composite event compared to those with lower HRs (70 or 71 bpm). For every 1 bpm increase in baseline HR, there was a corresponding 3% increase in the risk of adverse outcomes. These findings provided the justification for FDA approval of ivabradine for children with dilated cardiomyopathy and symptomatic HF after a single, randomized, double-blind, placebo-controlled trial.^58^ The study revealed that 70% of pediatric participants achieved the primary goal of a 20% reduction in HR from baseline without causing bradycardia or related symptoms. Despite no significant impact on traditional HF measures, such as NT-proBNP, HF classification, or quality of life, approval was granted. This was further supported by a multicenter registry study, which found that higher HRs were independently associated with increased mortality (adjusted HR 2.6) and the combined outcome of mortality or heart transplant (adjusted HR 1.5) in pediatric patients with HF.^59^

### Practical considerations for the use of oral medical therapies in pediatric heart failure

Given the heterogeneity of pediatric HF, it is difficult to make uniform recommendations regarding the use of HF therapies. However, there is likely sufficient evidence in pediatric patients >40 kg who have dilated cardiomyopathy and symptomatic (NYHA class II or higher) HF with reduced EF (LVEF <45%), to support a treatment paradigm that models adult HF guidelines. Dosing suggestions and cost-effectiveness for larger pediatric patients are included in Table 1; these are guidelines that should be individualized to patient-specific factors and end organ function.

**Table 1:** Suggested Dosing and Cost-effectiveness of Pediatric Heart Failure Therapies in Larger (>40 kg) Patients

Abbreviations: AA, aldosterone antagonist; ACEi, angiotensin-converting enzyme inhibitor; ARB, angiotensin receptor blocker; ARNI, angiotensin receptor/neprolysin inhibitor; BB, beta blocker; BID, twice daily; eGFR, estimated glomerular filtration rate; HCNCB, hyperpolarization-activated cyclic nucleotide-gated channel blocker; HR, heart rate; kg, kilograms; mg, milligrams; QALY, quality-adjusted life year; SGLT2i, sodium-glucose cotransporter 2 inhibitor; TID, three times a day; UA, urinalysis; yrs, years.

^a^Cost per quality-adjusted life year; $50-100,000 per QALY is accepted as a threshold in many cost-effectiveness analyses.

^b^There should be a 36-hour washout when switching between ACEi and ARNI to reduce the risk of angioedema.

^c^Do not use in the setting of hyperkalemia and/or eGFR <30 ml/min/1.73 m^2^.

^d^Recommend choice of SGLT2i based on what is on formulary with the insurance company.

In the Cost per QALY column, values highlighted in green, yellow, and red represent high, moderate, and low cost effectiveness, respectively.

In infants, we recommend a combination of captopril and carvedilol dosed 3 times a day along with an aldosterone antagonist, as first-line therapy. Dosing recommendations are included in Table 2. Due to immature renal function in young infants, close attention should be paid to serum electrolytes and estimated renal clearance with initiation and escalation of therapies. For patients with concomitant right ventricular failure and/or poor tolerance of beta-blockers, digoxin should be considered as adjunctive therapy. In patients with refractory tachycardia despite maximal beta blockade, the addition of ivabradine is reasonable; the dose should be increased every 2 weeks to achieve a 20% reduction in HR. We recommend caution with the use of SGLT2i and sacubitril-valsartan in infants given the absence of pharmacokinetic/pharmacodynamic and safety data in infants for the former, and the risk of hypotension with the latter. Furthermore, very few infants were enrolled in the PANORAMA-HF trial, leading to FDA approval of the drug only for patients >1 year old.

**Table 2:** Suggested Dosing of Pediatric Heart Failure Therapies in Small Patients

Age (years)	Class	Drug	Initial dose (mg/kg/dose)	Frequency	Titration amount (mg/kg/dose)	Time to dose increase	Goal dose
<1	ACEi	Captopril	0.05	TID	0.05-0.2	3-7 days	1 mg/kg/dose TID
	BB	Carvedilol	0.05	TID	0.05-0.1	14 days	0.5 mg/kg/dose TID
	AA	Spironolactone^a^	0.25	Daily	–	–	0.5 mg/kg/dose daily
	DG	Digoxin	0.005-0.006	BID	–	–	Target level 0.5-0.9 ng/ml
1-6	ACEi	Enalapril	0.05-0.1	BID	0.05-0.1	3-7 days	0.25 mg/kg/dose BID
	ARNI	Sacubitril-valsartan^b^	0.2	BID	0.2-0.4	3-14 days	3.1 mg/kg BID
	BB	Carvedilol	0.05	BID	0.05-0.1	14 days	0.5 mg/kg/dose TID
	HCNCB	Ivabridine	0.02-0.05	BID	0.05	14 days	20% heart rate reduction
	AA	Spironolactone^b^	0.5	Daily	--	--	0.5 mg/kg/dose daily
	DG	Digoxin	0.004-0.005	BID	--	--	Target level 0.5-0.9 ng/ml
	SGLT2i	Dapagliflozin^c^	0.1-0.2	Daily	0.1	UA for glucosuria 1-2 weeks after initiation/increase	Dose that results in 2+ glucosuria Max dose: 0.3 mg/kg/day, OR 5 mg in patients <30 kg, OR 10 mg in patients >30 kg
		Empagliflozin^c^	0.1-0.2	Daily	0.1	UA for glucosuria 1-2 weeks after initiation/increase	Dose that results in 2+ glucosuria Max dose: 0.3 mg/kg/day, OR 5 mg in patients <30 kg, OR 10 mg in patients >30 kg
>6 + <40 kg	ACEi/ARB	Enalapril	0.05-0.1	BID	0.05-0.1	3-7 days	0.25 mg/kg/dose ID
		Lisinopril	0.1	Daily	0.1	7 days	0.4 mg/kg/dose
		Losartan	0.7	Daily	0.3	7 days	No established dosing for ped HF; Max dose: 1.4 mg/kg/day
	ARNI	Sacubitril-valsartan^b^	0.2	BID	0.2-0.4	3-14 days	3.1 mg/kg BID
	BB	Carvedilol	0.05	BID	0.05-0.1	14 days	0.5 mg/kg/dose TID
		Metoprolol	0.25	BID	0.1-0.25	14 days	0.5 mg/kg/dose BID
	HCNCB	Ivabridine	0.02-0.05	BID	0.05	14 days	20% heart rate reduction
	DG	Digoxin	0.0025-0.004	BID	–	–	Target level 0.5-0.9 ng/ml
	AA	Spironolactone^a^	0.5	Daily	–	–	0.5 mg/kg/dose daily
	SGLT2i	Dapagliflozin^c^	0.1-0.2	Daily	–	–	5 mg in patients <30 kg 10 mg in patients >30 kg
		Empagliflozin^c^	0.1-0.2	Daily	–	–	5 mg in patients <30 kg 10 mg in patients >30 kg

Abbreviations: AA, aldosterone antagonist; ACEi, angiotensin-converting enzyme inhibitor; ARB, angiotensin receptor blocker; ARNI, angiotensin receptor/neprolysin inhibitor; BB, beta blocker; BID, twice daily; DG = digitalis glycoside; eGFR, estimated glomerular filtration rate; HCNCB, hyperpolarization-activated cyclic nucleotide-gated channel blocker; mg/kg/dose = milligrams per kilogram per dose; OR, odds ratio; QALY, quality-adjusted life year; SGLT2i, sodium-glucose cotransporter 2 inhibitor; TID, three times a day; UA, urinalysis; yrs, years.

a Do not use in the setting of hyperkalemia and/or eGFR <30 ml/min/1.73 m^2^.

b There should be a 36-hour washout when switching between ACEi and ARNI to reduce the risk of angioedema. The suggested dose refers to the combined dose of sacubitril and valsartan.

c Recommend choice of SGLT2i based on what is on formulary with the insurance company.

For children between 1 and 6 years old, it is reasonable to initiate BID dosing of carvedilol and enalapril, in addition to an aldosterone antagonist, as first-line therapy. We recommend the same considerations for the use of digoxin, ivabradine, and SGLT2i as in infants. It is reasonable to consider the use of sacubitril-valsartan as a second-line agent for those with refractory, symptomatic left ventricular dysfunction despite 6 to 12 months of standard therapy. Given the risk of hypotension in young patients, we recommend considering a more conservative approach to the initiation and escalation of sacubitril-valsartan.^42^

For children older than 6 years old and less than 30 kg, it is reasonable to consider SGLT2i and sacubitril-valsartan as first-line therapies, in addition to a beta blocker and aldosterone antagonist. For all patients, we recommend frequent (every 2-4 weeks) outpatient visits to achieve goal doses of HF therapies. A basic metabolic panel (BMP) should be obtained 1 to 2 weeks after initiation, and with every other increase in dose of an ACEi, ARB, sacubitril-valsartan, or SGLT2i. Once goal doses have been attained, a surveillance BMP and BNP should be obtained at least every 6 months for stable patients.

### Future Directions

There are several promising pediatric-specific drug trials underway that incorporate pharmacokinetic/pharmacodynamic testing in children of various ages and will provide crucial data on safety and efficacy in children.

#### Soluble guanylate cyclase stimulator

Under normal conditions, nitric oxide (NO) binds to soluble guanylate cyclase, an enzyme that catalyzes the synthesis of intracellular cyclic guanosine monophosphate, a second messenger that plays a role in the regulation of vascular tone, cardiac contractility, and cardiac remodeling. HF is associated with a decrease in the synthesis of NO.^60^ Vericiguat directly stimulates soluble guanylate cyclase and separately potentiates the effect of endogenous NO, leading to smooth muscle relaxation and vasodilation. In adults with reduced EF who have already experienced an acute HF exacerbation, the addition of Vericiguat to guidelines-directed medical therapy resulted in decreased HF hospitalizations (27.4% vs 29.6%) and death from cardiovascular causes (16.4% vs 17.5%) compared to those receiving placebo. The medication has intermediate cost-effectiveness. The VALOR trial is currently enrolling participants aged 29 days to 17 years to study the pharmacokinetics/pharmacodynamics, safety, and efficacy of Vericiguat in pediatric patients. The target enrollment is 342 patients and the primary end-point is change in NT-proBNP at 16 weeks. Secondary outcomes include time to first cardiovascular event (cardiovascular death, hospitalization, or worsening HF) and change in log-formatted NT-proBNP, at 52 weeks.^61^

#### Cardiac myosin inhibitors

HCM carries a high burden of morbidity and mortality in children and adults. Historically, for patients with symptomatic, obstructive HCM, options included beta- or calcium channel-blockade with questionable efficacy and variable tolerability, or invasive procedures such a surgical myectomy or alcohol septal ablation that carry a risk of heart block and infection. The development of cardiac myosin inhibitors has revolutionized the field for patients with obstructive HCM, resulting in regression of septal hypertrophy, decreased obstruction, and improvement in HF symptoms with the use of mavacamten^62^, ^63^; and improved peak oxygen consumption with aficamten.^64^ Both drugs are being studied in adolescents with obstructive HCM. The primary efficacy end-point for both the SCOUT-HCM trial of mavacamten^65^ and the CEDAR-HCM trial of aficamten^66^ is a decrease in the left ventricular outflow gradient by echocardiogram during a Valsalva maneuver. This can be used as a surrogate for the symptomatic improvement and decrease in NT-proBNP and high-sensitivity troponin observed in adult studies.

### Gene therapy

Gene therapy is promising and offers hope for rare, devastating diseases such as Danon disease and DMD. Danon disease is a rare, autosomal dominant, X-linked disease caused by a LAMP-2B gene mutation resulting in impaired autophagy that places male patients at risk for cardiomyopathy and mortality in their 20s to 30s.^67^ Rarely, female patients may also be effected. Enrollment has been completed for a phase 2 study that introduces a functional LAMP-2 gene via a viral vector; the primary outcome measure is gene expression and change in serum troponin.^68^ DMD is a progressive, degenerative muscle disease affecting 1 in 3,500 to 5,000 births. It is caused by a defect in the DMD gene, resulting in the lack of the protein dystrophin, which leads to the breakdown of muscle fibers and replacement by fibrous or fatty tissue, and ultimately severe muscle weakness and early demise in the 20s to 30s. The FDA granted accelerated approval to the gene therapy Delandistrogene moxeparvovec (Elevidys) for DMD after 2 small trials of ambulatory boys >4 years old with DMD revealed improvement in the North Star Ambulatory Assessment Score, and evidence of microdystrophin expression on muscle biopsy.^69^ After approval, the EMBARK trial, a phase 3 randomized trial of Delandistrogene moxeparvovec vs placebo, both of which were administered once via an adenovirus viral vector, revealed no difference between the groups in North Star Ambulatory Assessment Score at 52 weeks of follow-up.^70^ There were no deaths, discontinuations, or significant complement-mediated adverse events; 7 patients (11.1%) experienced 10 treatment-related serious adverse events that were manageable.

## Conclusion

Despite advancements in the treatment of pediatric HF, the diversity of underlying causes and the relative rarity of the condition pose unique challenges to developing effective treatments. Recent initiatives have accelerated the availability of pediatric-specific therapies, but more targeted research is needed to ensure that children with HF receive therapies that are both safe and effective for their unique physiology.

## CRediT authorship contribution statement

All others have contributed to conceptualization, resource acquisition, methodology, writing, and reviewing the document.

## Disclosure statement

The authors declare the following financial interests/personal relationships which may be considered as potential competing interests: Joseph Rossano reports a relationship with Merck and Co. Inc. that includes consulting or advisory. Joseph Rossano reports a relationship with Bayer Corporation that includes consulting or advisory. Joseph Rossano reports a relationship with Bristol Myers Squibb Co. that includes consulting or advisory. Joseph Rossano reports a relationship with CRI Biotech that includes consulting or advisory. Joseph Rossano reports a relationship with Asklepios BioPharmaceutical Inc. that includes consulting or advisory. The other authors declare that they have no known competing financial interests or personal relationships that could have appeared to influence the work reported in this paper.
